# Neonatal bacteraemia in Ireland: A ten-year single-institution retrospective review

**DOI:** 10.1371/journal.pone.0306855

**Published:** 2024-08-23

**Authors:** James Powell, Irene Beirne, Brid Minihan, Nuala H. O’Connell, Santosh Sharma, Margo Dunworth, Roy K. Philip, Colum P. Dunne

**Affiliations:** 1 Department of Microbiology, University Hospital Limerick, Limerick, Ireland; 2 School of Medicine and Centre for Interventions in Infection, Inflammation and Immunity (4i), University of Limerick, Limerick, Ireland; 3 Department of Paediatrics, Division of Neonatology, University Maternity Hospital Limerick, Limerick, Ireland; 4 University of Limerick School of Medicine, Limerick, Ireland; 5 School of Pharmacy, Queen’s University Belfast, Belfast, Northern Ireland; Nitte University, INDIA

## Abstract

Neonatal sepsis is a catastrophic condition of global concern, with reported mortality rates exceeding 10%. Bloodstream infections are an important cause of sepsis, and epidemiological studies of these infections are crucial for predicting the most common aetiological agents and antimicrobial susceptibility patterns and for developing antimicrobial guidelines. For the ten-year study period from July 2013 to June 2023, all neonatal bacteraemia cases were reviewed prospectively using an enhanced surveillance protocol. The patients were stratified according to their age at the time of blood culture collection: early onset if diagnosed in the first 72 hours of life, and late onset if diagnosed after that time. During the study period, 170 blood cultures were positive from 144 patients, of which 89 specimens from 64 patients represented the growth of significant pathogens. Coagulase-negative *staphylococci* (CoNS) were the most common pathogens identified (52%, 33/64), followed by *Escherichia coli* (14%, 9/64), Group B *Streptococcus* (GBS: 11%, 7/64) and *Staphylococcus aureus* (11%, 7/64). GBS was more commonly identified in early onset patients, while CoNS were predominantly associated with late onset. The presence of an intravascular catheter, maternal urinary tract infections and the receipt of total parenteral nutrition or transfused blood were identified as significant risk factors. The fatality rate was 8% (5/64). in summary, this study provides a detailed overview of the epidemiology of neonatal bacteraemia in a large teaching hospital in the Midwest of Ireland over a decade.

## Introduction

Reducing neonatal mortality is a matter of global importance [1]. The neonatal mortality rate was defined as 28-day mortality per 1,000 live births. It has been in decline from 1990 to 2021 globally (36.6 to 17.6), in Western Europe (5.5 to 2.2) and in Ireland (4.7 to 2.1) [2]. The highest rates of neonatal mortality are evident in West and Central Africa (30.5) and Sub-Saharan Africa (27.1) [2], contemporary rates that exceed the highest recorded historical rates in Ireland (21.2, from the earliest available data in 1960, a time when many babies were born in ‘maternity homes’ with limited timely paediatric care) [3].

Sepsis occurs when an infection exceeds local tissue containment and induces a series of dysregulated physiological responses, resulting in organ dysfunction [4], and bloodstream infections are an important cause of sepsis [5]. Meta-analyses of neonatal sepsis have shown pooled mortality rates of 10.9% [6] and 17.6% [7]. The diagnosis of sepsis in neonates is challenging given the nonspecific signs and symptoms of the disease [8]. Blood cultures provide crucial information for sepsis management; however, test sensitivity is reduced by limited blood volume from neonates and the effect of maternal antimicrobial therapy [9]. A common practice is to start empiric antibiotics until sepsis is proven or excluded [8]. This can lead to the widespread and prolonged use of antibiotics, as blood culture results can take several days to become available. Therefore, epidemiological studies are crucial for predicting the most common aetiological agents and antimicrobial susceptibility patterns and for developing antimicrobial guidelines [8].

There is no overarching consensus on GBS screening protocols. In 2002, the Centers for Disease Control and Prevention issued guidelines recommending universal screening of pregnant women between 35 and 37 weeks gestation [10]; however, GBS-EONS continues to be an issue where this is practiced, with 60–80% of GBS-EONS babies born to mothers who were negative for GBS at that screen [11]. Despite this, a meta-analysis of neonatal outcomes revealed that the screening strategy for prophylaxis was superior to a risk-based approach [12]. National guidelines recommend a choice of options for GBS screening: a risk-based approach with or without real-time PCR testing or universal screening [13]. In our hospital, urine screening in the first trimester is almost universally conducted, vaginal swabs of pregnant patients are routinely screened upon receipt, and rectal screening for GBS is rarely performed (approximately 10% of mothers, local unpublished data). This was the case for the entirety of the study period, with no proposals for change.

Maternal risk factors for neonatal sepsis include prolonged rupture of membranes (PROM), premature prolonged rupture of membranes (PPROM), GBS colonisation and procedures performed during pregnancy [14]. Risk factors for EONS include lower birth weight and shorter gestational age [15], and the primary risk factor for LONS is central line catheterisation [16]. Preventive measures include site-specific quality improvement projects to ensure the maintenance of optimal hand hygiene, antimicrobial stewardship and the introduction of care “bundles” for line management [17].

Studying the epidemiology of neonatal sepsis is crucial for purposes such as tracking the responsible pathogens, providing guidance for both local and international empirical treatments, recognizing regional risk factors and interventions used to reduce them, and influencing the development of alternative solutions such as maternal vaccines [18]. The aim of this study was to characterise the microbial epidemiology and antimicrobial susceptibility of blood pathogens from the NICU of a large teaching hospital in Western Europe and to describe the risk factors and clinical features of those infections.

## Methods

Limerick University Maternity Hospital is the only maternity hospital in the Midwest of Ireland; in 2020, it had a population of 488,300 people [19] and 4069 live births [20]. Previous relevant research from this institution included a review of time-to-positivity (TTP) of neonatal blood cultures [21], an interventional study for reducing blood culture contamination in neonates [22], and reports of neonatal and other outbreaks [23–25]. This study was approved by the Research Ethics Committee of the University of Limerick Hospital Group, Limerick, Ireland (Ref 037/2023). The ethics committee can be contacted at ULHGResearchEthicsandClinicalTrials@hse.ie and https://www.ul.ie/hsa/innovation-and-research/clinical-research-ethics-ul-hospital-group-and-mid-west-community.

For the ten-year study period from July 2013 to June 2023, consecutive paediatric FAN® Plus aerobic blood cultures were incubated for up to five days on the BACT/ALERT® 3D (bioMerieux, Marcy-l’Étoile, France) system. Blood cultures and other diagnostic specimens were collected according to National Institute for Health and Clinical Excellence (NICE) guidelines [26] according to the criteria of one red flag or two nonred flag risk factors or clinical indicators. The cases were reviewed prospectively by both a senior clinician and a nurse manager (see S1 and S2 Figs for surveillance forms). The classification of isolates as contaminants or significant was made in conjunction with clinical microbiologists, taking into account the symptomatology of the patient and the organism identification. Cerebrospinal fluid and non-culture (molecular) microbiology results were not included in this study, and patients admitted to other hospitals in the network were also excluded. Neonatal sepsis is sometimes classified as Early Onset Neonatal Sepsis (EONS) if diagnosed in the first seven days of life [27] but a more common cut-off is the first 72 hours [28,29] and this definition was used for our study. Term infants were defined in our study according to the World Health Organisation classification [30]. The data were anonymised before analysis; summary statistics were generated using Microsoft Excel 2016 and further statistical analysis was performed using STATA/SE 17.0 version. We used t tests, chi-square tests, Fisher’s exact tests and binary logistic regression analyses to compare risk factors.

## Results

During the study period, 42,174 births (42,996 infants) were recorded, which comprised 98.1% (n = 41,366) singletons, 1.9% (n = 794) twins and 14 (< 0.1%) other multiples. A total of 210 stillborn infants were recorded, comprising 82.4% (n = 173) singletons, 15.2% (n = 32) twins and 2.4% (n = 5) other multiples. In the same period, 8,490 (20%) infants were admitted to the neonatal unit; 86% (n = 7,327) were admitted on their first day of life, and 99.6% (n = 8,460) were admitted within the first week. The median length of stay was 3 days (IQR 1–7 days).

**Fig 1:**
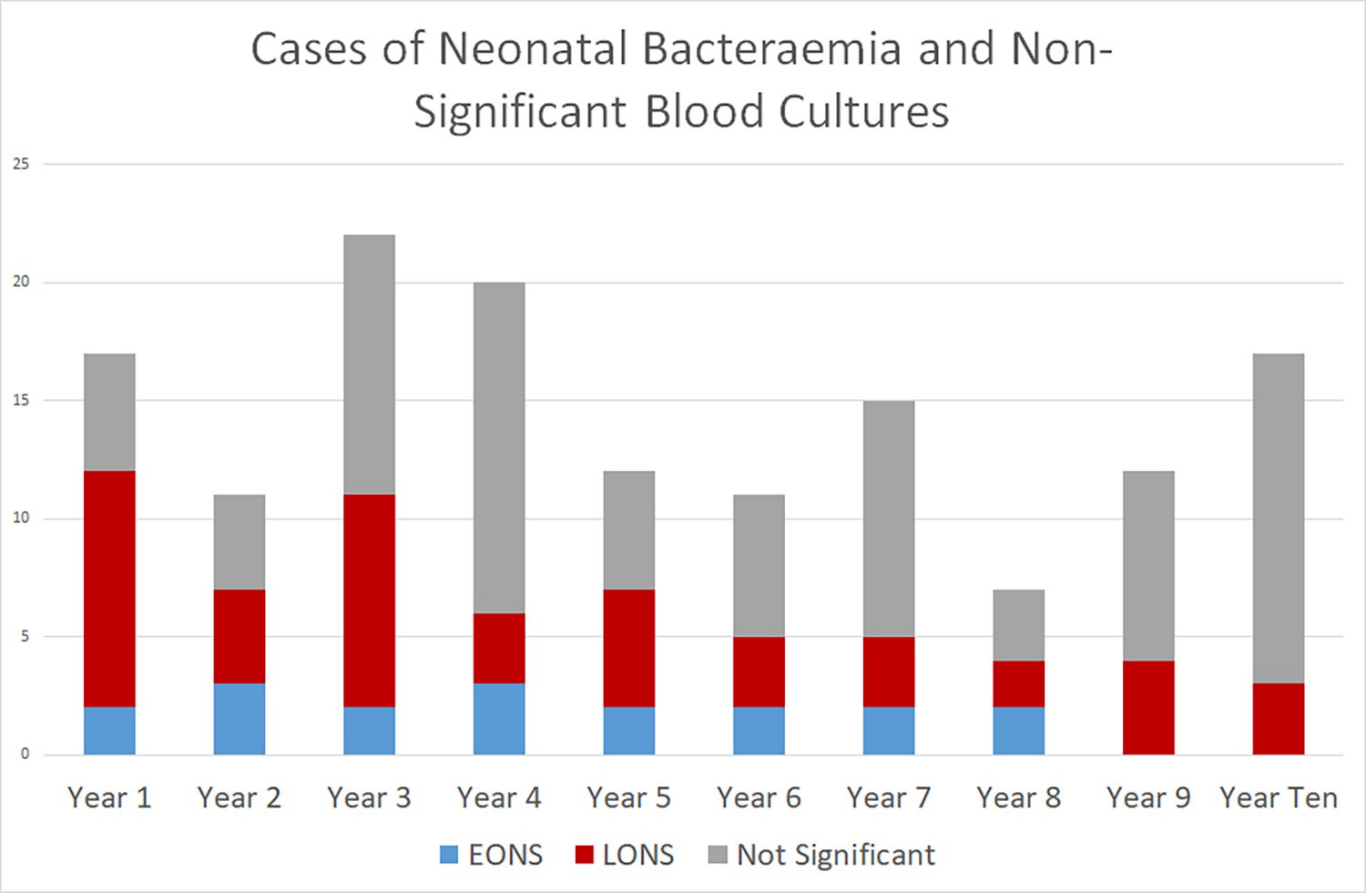
Number of cases of neonatal bacteraemia per year, early onset neonatal sepsis (EONS, < 72 hours of age), late onset neonatal sepsis (LONS, > 72 hours of age) and “not significant” (positive blood cultures yielding growth not considered significant, i.e., contaminants).

A total of 170 blood cultures were positive from 144 patients, of which 89 specimens from 64 patients represented the growth of significant pathogens. A total of 99% (142/144) of the infants were admitted to the neonatal unit shortly after birth without discharge from the hospital; two infants were admitted from home or another centre, and both were positive according to specimens collected more than 48 hours postadmission. Therefore, by definition, all the cases were hospital-acquired [15]. The median length of stay for EONS patients was 11 days (I.Q.R. 3 days– 21 days), 52 days for LONS patients (I.Q.R. 25 days– 85 days) and 10 days for those with nonsignificant cultures (I.Q.R. 4 days– 63 days). A reduction in cases of both EONS and LONS was noted: 7 EONS and 23 LONS in the first three years of the study, compared with two EONS and 9 LONS cases in the final three years of the study. The incidence of contamination in the cultures fluctuated but remained below 3% throughout the study period, with no observable temporal trend (Fig 1).

**Fig 2:**
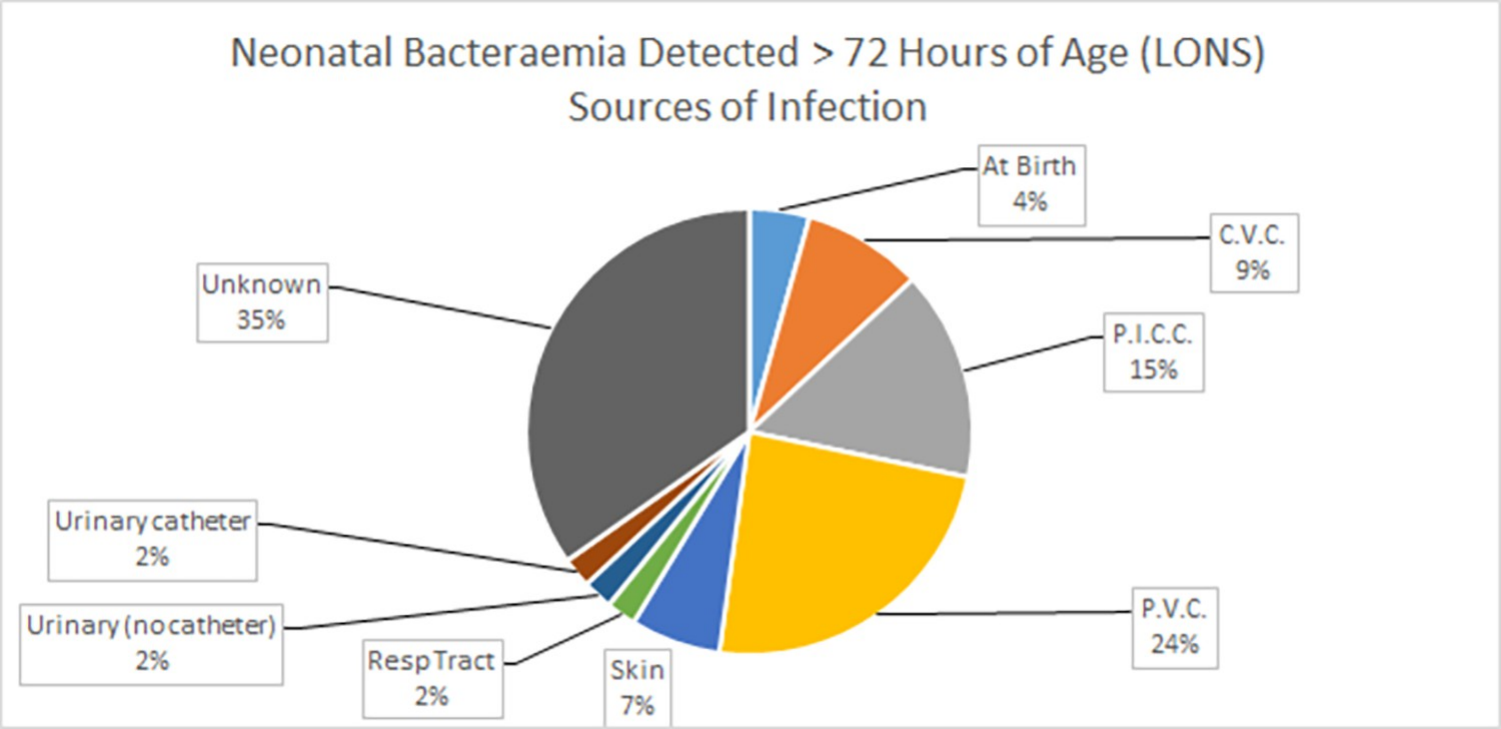
Source of infection: Patients > 72 hours of age (LONS).

**Fig 3:**
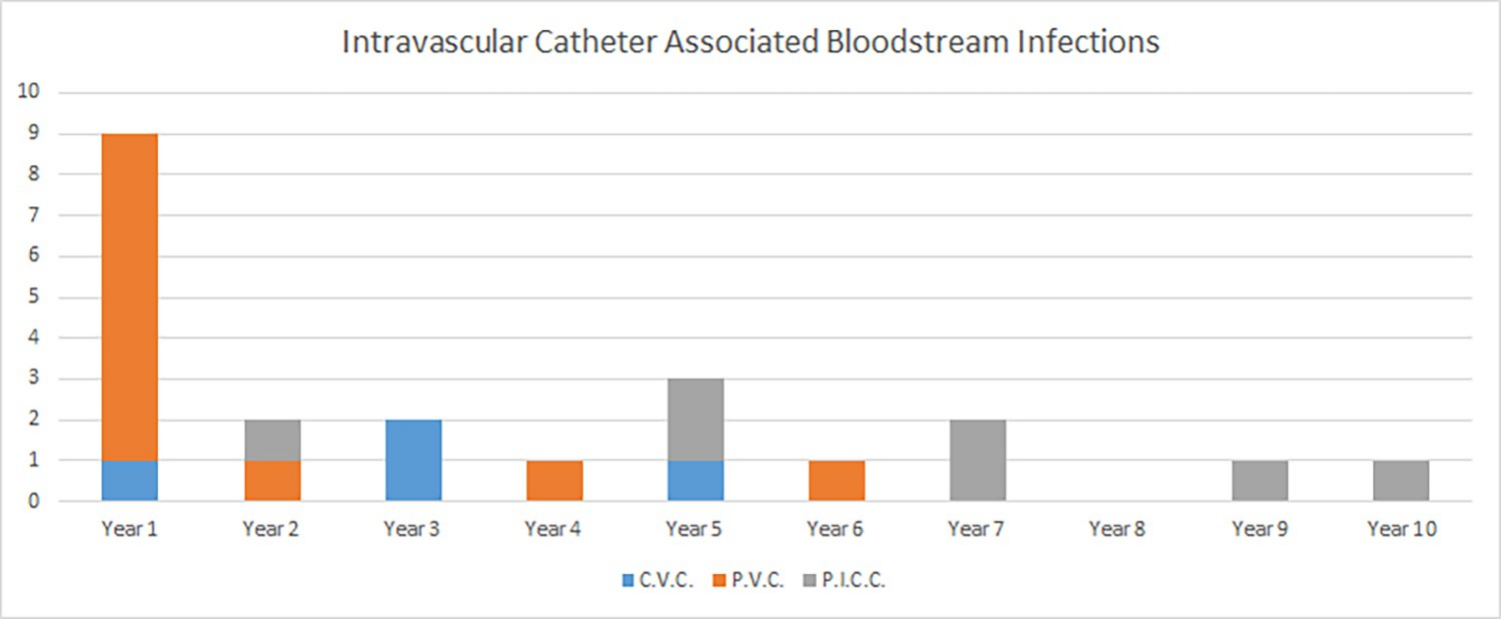
Intravascular catheter-associated infections per year Central venous catheter (CVC), peripheral venous cannula (PVC) and peripherally inserted central catheter (PICC).

The sources of infection for EONS patients were exclusively “at birth” (n = 12) or “unknown” (n = 6). The primary sources for the LONS patients were intravascular catheters (n = 22) and “unknown” (n-16) (Fig 2). There was a reduction in some intravascular catheter-associated cases (see Fig 3): ten peripheral vascular catheter (PVC)- and four central line (CVC)-associated cases in the first half compared to one PVC and zero CVC cases in the second half. Conversely, there were three peripherally inserted central catheter (PICC)-associated cases in the first half of the study and four cases in the second half. The incidence rates of PICC-related line infections were influenced by increased usage in the second half of the study, in line with international reports [31].

CoNS represented the majority (91/144, 63%) of the bloodstream organisms identified; *Staphylococcus epidermidis* (44%, 35/79) and *Staphylococcus capitis* (22%, 17/79) were the most common species (of those that were identified; see S3 Table). Beta haemolytic *Streptococcus* group B (GBS) accounted for 33% (6/18) of the EONS cases (0.16 cases per 1,000 live births), four of which were serotype III (one serotype Ia, one unknown). GBS was also identified from a LONS (12 days of age) patient (serotype III). All seven mothers were screened for GBS from urine samples in their first trimester, and no GBS was detected. *Escherichia coli* accounted for 33% (6/18) of the EONS patients. CoNS (67%, 31/46), followed by *Staphylococcus aureus* (n = 7), were responsible for the greatest number of LONS cases (Table 1). *E*. *coli* was more prevalent in the first half of the study than in the second half (6 patients vs 3 patients); however, the reverse was observed for other Enterobacterales (0 patients vs 3 patients). Three cases of enterococcal bacteraemia were recorded in the first half compared to zero cases in the second half, and 55 cases of staphylococcal bacteraemia (five *S*. *aureus*) versus 42 cases (two *S*. *aureus*) were recorded in the second half. One case of *Candida albicans* fungaemia was detected.

**Table 1:** Causative organisms (clinically significant) of bacteraemia cases, early (EONS) and late (LONS) onset neonatal sepsis.

Organism	EONS	LONS	Total
*Escherichia coli*	6	3	9
Beta Haemolytic Strep.Gp.B	6	1	7
*Staphylococcus aureus*	0	7	7
*Enterococcus* species	1	1	2
*Candida albicans*	0	1	1
*Enterobacter cloacae*	1	0	1
*Klebsiella pneumoniae*	0	1	1
*Proteus mirabilis*	0	1	1
*Haemophilus influenzae*	1	0	1
*Streptococcus pneumoniae*	1	0	1
*Streptococcus oralis*	1	0	1
Coagulase Negative Staph Gp.	2	31	33
Total	18	46	64

The median gestational age was 29 weeks for the LONS patients and 37 weeks for the EONS patients. A younger gestational age was a significant risk factor (p<0.005) for EONS and LONS (combined); as gestational age increased, the odds of neonatal sepsis (EONS and LONS) were significantly lower (OR = 0.99, 95% CI = 0.979, 0.996). Neither patient gender (p = 0.6) nor the presence of a congenital abnormality (p = 1.0) was a significant risk factor. Intravascular catheters were a significant risk factor (p<0.005, OR = 3.04, 95% CI = 1.41, 6.57). There was no statistical significance for UAC, UVC or PVC catheters individually, but PICC lines were found to be a significant risk factor (p<0.005, OR = 3.07, 95% CI = 1.48, 6.36). Total parenteral nutrition (TPN, with or without lipids, p<0.005; OR: 3.77, 95% CI 1.45, 9.79), blood transfusion (p<0.05, OR: 3.82, 95% CI 1.28, 11.38) and maternal UTI (p<0.001) were also identified as significant risk factors. The following risk factors were not significantly associated with bacteraemia in our patients: Prolonged rupture of membranes (PROM) (p = 0.36), maternal fever (p = 0.82), the use of ventilation (p = 0.11) or the presence of an endotracheal tube (p = 0.13); see Tables 2 and S2–S4. The clinical features of the bacteraemia cases are summarised in Table 3.

**Table 2:** Risk factors for acquiring early (EONS) and late (LONS) onset neonatal bacteraemia.

Risk Factor	EONS No. (%, n = 18)			LONS No. (%, n = 46)			Non-Significant No. (%, n = 80)		p value	Odds Ratio (95% C.I.)
Male gender	13	(72.2%)		25	(54.3%)		44	(55.0%)	0.370	1.20 (0.61, 2.33)
Preterm	9	(50.0%)		43	(93.5%)		49	(61.3%)	<0.001	2.71 (1.27, 5.93)
PROM	6	(33.3%)		10	(21.7%)		15	(18.8%)	0.400	1.44 (0.65, 3.21)
Maternal fever	6	(33.3%)		2	(4.3%)		9	(11.3%)	0.005	1.13 (0.41, 3.11)
Maternal UTI	3	(16.7%)		7	(15.2%)		0	(0.0%)	<0.001	N/A^1^
P.I.C.C.	5	(27.8%)		24	(52.2%)		17	(21.3%)	0.001	3.07 (1.48, 6.36)
P.V.C.	8		(44.4%)	26		(56.5%)	35	(43.8%)	0.370	1.46 (0.75, 2.82)
U.A.C.	4		(22.2%)	10		(21.7%)	13	(16.3%)	0.690	1.44 (0.62, 3.34)
U.V.C.	5	(27.8%)		12	(26.1%)		11	(13.8%)	0.150	2.27 (0.98, 5.28)
Intravascular line (any)	6	(33.3%)		39	(84.8%)		47	(58.8%)	0.004	3.04 (1.41, 6.57)
ETT	4	(22.2%)		10	(21.7%)		10	(12.5%)	0.320	1.96 (0.81, 4.77)
Ventilated	3	(16.7%)		21	(45.7%)		20	(25.0%)	0.021	1.80 (0.88, 3.68)
TPN/Lipids	2	(11.1%)		15	(32.6%)		7	(8.8%)	0.002	3.77 (1.45, 9.79)
Blood transfused	2	(11.1%)		11	(23.9%)		5	(6.3%)	0.021	3.82 (1.28, 11.38)
Congenital abnormality	0	(0.0%)		1	(2.2%)		1	(1.3%)	1.000	1.25 (0.08, 20.45)

^1^ Not applicable; no maternal UTI cases were included in the control group, so the odds ratio could not be calculated. Abbreviations: Prolonged rupture of membranes (PROM). Total parenteral nutrition (TPN). Umbilical arterial catheter (UAC), umbilical venous catheter (UVC), peripheral venous cannula (PVC) and peripherally inserted central catheter (PICC).

**Table 3:** Clinical features of early (EONS) and late (LONS) onset neonatal bacteraemia patients.

Clinical Features	EONS (n = 18)		LONS (n = 46)	
Fever	8	(44.4%)	20	(43.5%)
Temperature instability	1	(5.6%)	8	(17.4%)
Hypothermia	2	(11.1%)	1	(2.2%)
Tachycardia	2	(11.1%)	5	(10.9%)
New/Increased bradycardia	2	(11.1%)	15	(32.6%)
New onset hyperglycaemia	4	(22.2%)	10	(21.7%)
Hypoglycaemia	1	(5.6%)	3	(6.5%)
Leukocytes <5 leuk/nl	4	(22.2%)	3	(6.5%)
Leukocytes >23 leuk/nl	0	(0.0%)	3	(6.5%)
Platelets <100 plts/nl	2	(11.1%)	4	(8.7%)
Hypotension	1	(5.6%)	3	(6.5%)
CRT>2	3	(16.7%)	11	(23.9%)
CRP>2 mg/dl	5	(27.8%)	14	(30.4%)
New/increased apnoea	1	(5.6%)	14	(30.4%)
Unexplained metabolic acidosis	2	(11.1%)	1	(2.2%)
Unstable general condition	4	(22.2%)	14	(30.4%)
Apathy/lethargy	2	(11.1%)	15	(32.6%)
Apnoea	1	(5.6%)	8	(17.4%)
Pneumonia	1	(5.6%)	0	(0.0%)
Feeding intolerance	1	(5.6%)	3	(6.5%)
Omphalitis	0	(0.0%)	0	(0.0%)

Antimicrobial resistance was not prevalent among our bacterial isolates; 33% (4/12) of the Enterobacterales strains had no detectable antimicrobial resistance, and with the exception of one isolate of *Escherichia coli*, which was resistant to ampicillin, coamoxiclav and trimethoprim-sulfamethoxazole. All of the remaining Enterobacterales strains showed resistance to just one class of antimicrobial agents (the β-lactam class, including one Amp C producer and one ESBL producer). The susceptibility of coagulase-negative staphylococci to flucloxacillin was 18% (6/33), and 100% were susceptible to vancomycin. All (7/7) of the *Staphylococcus aureus* isolates were susceptible to flucloxacillin. All of the streptococci and enterococci were susceptible to β-lactam and glycopeptide antimicrobial agents; although 29% (2/7) of the GBS isolates were resistant to clindamycin, 43% (3/7) were resistant to erythromycin, and 86% (6/7) were resistant to tetracycline.

Six (4%, n = 144) of the neonates died during admission or within 30 days of discharge; three of whom were EONS patients, two were LONS patients, and one had nonsignificant cultures (17%, 4% and 1% of each cohort). Seventeen patients were transferred to another hospital (12%, n = 144): 4 had EONS, three had LONS, and ten had nonsignificant cultures (22%, 7% and 13% of each cohort). The remaining 121 patients (84%, n = 144) were discharged home (61%, 89% and 86% of the EONS, LONS and nonsignificant culture patients, respectively). Three of the six infants who died had Gram negative bacteraemia (*E*. *coli* X 2, *E*. *cloacae*).

## Discussion

The bacteraemia rate in our study was only 1.5 cases per 1,000 live births and 0.4 EONS cases per 1,000 live births or 2 cases per 1,000 admissions. Bacteraemia frequently arises as a complication of necrotizing enterocolitis (NEC) in infants [32]. The incidence of NEC at University Maternity Hospital Limerick our hospital is among the lowest in Ireland [33], which is consequentially associated with a reduced occurrence of bloodstream infections. The low NEC rate has been primarily credited to the successful introduction of a quality improvement project, which resulted in a 100% human milk exposure for extremely low birth weight infants [33]. The 20% admission rate in our unit is reflected by a low threshold for admission: All infants under 35 weeks of gestation or with a bodyweight less than 2.2 kg are admitted. Those weighing between 2.2 kg and 2.5 kg undergo clinical assessment and are admitted if there are any concerns. The majority of infants requiring antimicrobial agents are admitted unless antimicrobial prescribing is based only on maternal risk factors (such as GBS carriage, PROM > 18 hours, maternal pyrexia > 38°) and if the baby is over 35 weeks of gestation, 2.5 kg bodyweight and is clinically healthy.

The organisms identified are typical of high-income nations: CoNS are responsible for approximately 50% of neonatal primary bloodstream infections in industrialised nations [34], which was also the case in our study (50%, 32/64). Gram-negative rods are more common in the developed world than in the developing world, as is antimicrobial resistance [35]. Antimicrobial resistance was not prevalent among the bacteria in this study, with almost all the isolates showing susceptibility to first-line antimicrobial agents. For GBS, substantial clindamycin resistance (29%) supports the decision to use vancomycin prophylaxis for penicillin-allergic patients.

The first-line empirical therapy in our unit for EONS is benzylpenicillin (amoxicillin if *Listeria monocytogenes* is suspected and cefotaxime if meningitis is suspected) and gentamycin. For LONS, flucloxacillin (or alternatively vancomycin if line sepsis is strongly suspected) and gentamicin are used. Decisions regarding second-line treatment options and antimicrobial switching are made by neonatal clinicians collaboratively with the involvement of the clinical microbiology team, guided by culture results and clinical response to treatment. Meropenem is reserved for cases of treatment failure or in cases of known maternal ESBL colonisation (all mothers of babies admitted to the NICU were screened for ESBL-producing enterobacterales). Fungal prophylaxis with fluconazole is given in certain circumstances, such as for extremely low birth weight (ELBW) infants. GBS prophylaxis for penicillin-allergic patients consists of clindamicin when susceptibility is established, vancomycin when susceptibility to clindamicin is unknown or if resistance is documented.

*Staphylococcus capitis* outbreaks have been reported globally [36], and this organism was the second most commonly detected CoNS in our study. However, whole-genome sequencing of these isolates was not performed to identify the problematic “NRCS-A” [37] strain. A significant proportion of the CoNS in our study were not identified however, so the exact incidence of *S*. *capitis* infection could not be determined. There were no obligate anaerobes detected in our study, which may be an artefact of the exclusive use of aerobic blood culture bottles in our hospital. We observed only a single case of fungaemia, which aligns with findings from other studies that have also reported low incidences of fungaemia [15] and can be credited to the use of prophylactic antifungal therapy (fluconazole).

Contaminated blood cultures are known to be detrimental to patient care [38]. The contamination rate in our study fluctuated between 1% and 2%, below the recommended threshold of 3% [39], but not within the more recent 1% target [40]. The case fatality rate for EONS (17%) was greater than that for LONS (4%), in accordance with previous reports [41]. Interventions used in our hospital to reduce the blood culture contamination rate include skin antisepsis via sterile applicators with 2% chlorhexidine gluconate in 70% isopropanol prior to phlebotomy (replacing 70% isopropanol) and staff education, which were all introduced in a quality improvement project [22]. Other infection prevention and control interventions that have been introduced in our hospital include care bundles for intravascular catheters, the use of a single catheter when possible, and prompt removal when no longer required. Several risk factors, such as low birth weight [27,42–44], low gestational age [15,27,43,45,46] and PROM [45–48], have been widely reported, but others, such as healthcare worker hand washing [47], birth outside of the hospital [42,45] and lack of parental financial assistance [47], have been reported only for individuals from developing nations. Our study identified maternal UTIs, PICC line catheterisations, and the receipt of TPN or transfused blood.

Some limitations apply to this study, which was performed without the assistance of an electronic patient record. Only patients with bloodstream culture-confirmed cases were included, and viral aetiologies were not examined. Birth weights were not recorded until the late stage of the study period and were not included. Neonates admitted to a multispecialty hospital in the regional network were excluded, so our true incidence rates may be underestimated. The COVID-19 pandemic took place in the latter part of our study period; No attributable epidemiological changes were observed, though some may have gone undetected.

## Conclusion

This study provides a detailed overview of the epidemiology of neonatal bacteraemia covering a decade and almost 43,000 live births in a large teaching hospital in the Midwest of Ireland. We describe the organisms identified and their antimicrobial resistance, offering essential direction for empirical therapy, and we have highlighted a number of modifiable risk factors. While the overall findings are consistent with prior reports from developed Western countries, our observation of a downwards trend in case numbers and low antimicrobial resistance levels among the identified pathogens suggest the benefits of adhering to infection prevention and control measures, enhanced antimicrobial surveillance, quality improvement projects targeting ‘neonatal aseptic clinical behaviours’ and antimicrobial stewardship.

## Supporting information

S1 Fig Enhanced Surveillance Form V1 (2013–2014). (TIF)

S2 Fig Enhanced Surveillance Form V8 (2018 –present). (TIF)

S1 Table Coagulase Negative Staphylococci identified. (DOCX)

S2 Table Neonatal bacteraemia early (EONS) and late (LONS) onset by background and risk factor. (DOCX)

S3 Table Results of binary logistic regression unadjusted and adjusted Odds ratios of Neonatal bacteraemia by background and risk factor. (DOCX)

S4 Table Results of binary logistic regression adjusted Odds ratios of neonatal bacteraemia by background and risk factor. (DOCX)

## Abbreviations

EONS: Early Onset Neonatal Sepsis
LONS: Late Onset Neonatal Sepsis
CoNS: Coagulase negative *Staphylococci*
GBS: Group B *Streptococcus*
PROM: Prolonged rupture of membranes
PPROM: Premature prolonged rupture of membranes
NICE: National Institute for Health and Clinical Excellence
PVC: Peripheral vascular catheter
CVC: Central venous catheter
PICC: Peripherally Inserted Central Catheter
TPN: Total parenteral nutrition
NEC: Necrotizing enterocolitis
ESBL: Extended Spectrum Beta-Lactamase
ELBW: Extremely low birth weight
