# Supplementary figures and images for: Neonatal bacteraemia in Ireland: A ten-year single-institution retrospective review
Source: PLoS One. 2024 Aug 23;19(8):e0306855. doi: 10.1371/journal.pone.0306855 (PMC11343407; doi:10.1371/journal.pone.0306855)

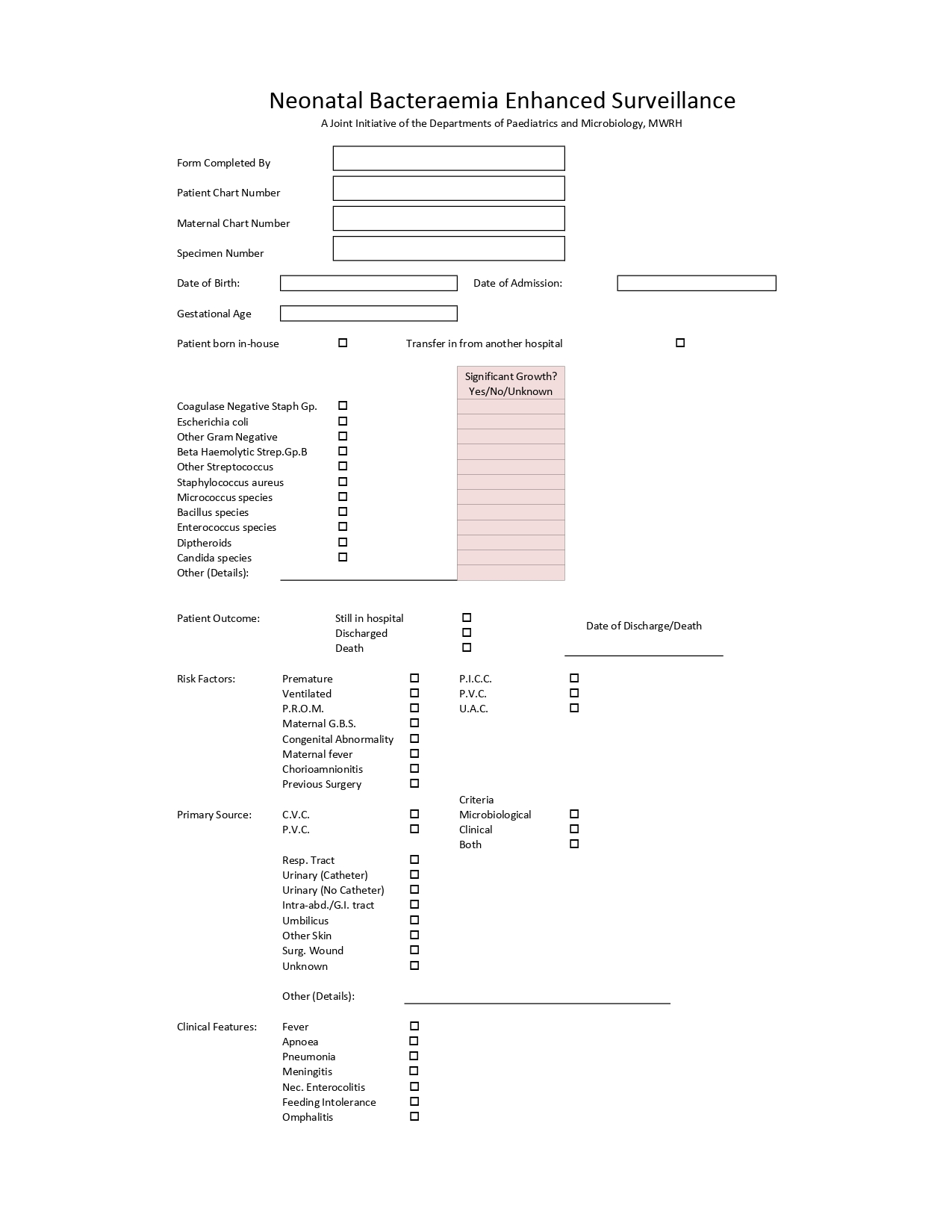

Supplement: S1 Fig — (TIF) [file pone.0306855.s001.tif]

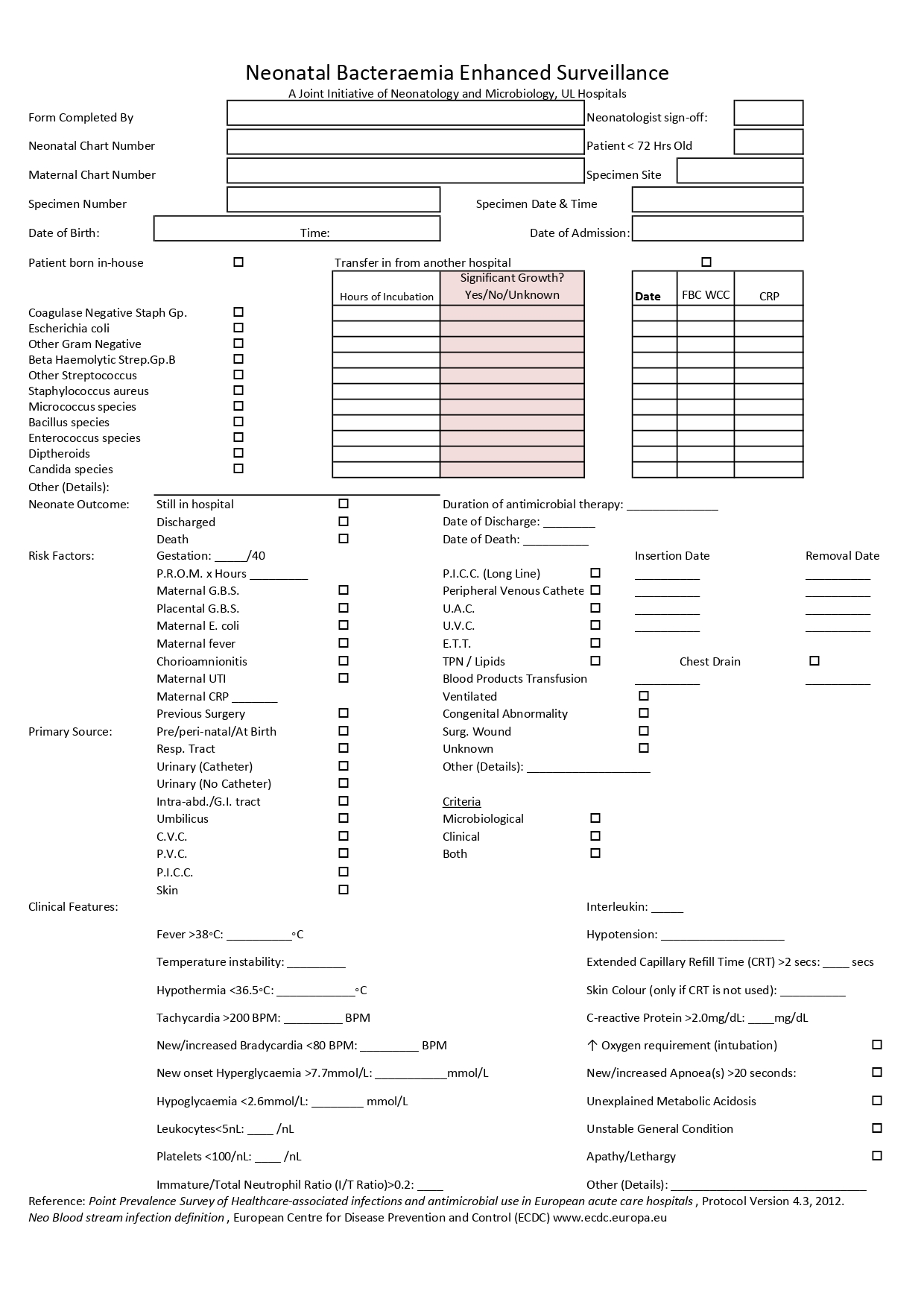

Supplement: S2 Fig — (TIF) [file pone.0306855.s002.tif]
