# Supplementary material for: Neonatal bacteraemia in Ireland: A ten-year single-institution retrospective review
Source: PLoS One. 2024 Aug 23;19(8):e0306855. doi: 10.1371/journal.pone.0306855 (PMC11343407; doi:10.1371/journal.pone.0306855)
Supplement: S1 Table — (DOCX) [file pone.0306855.s003.docx]

|  | Not Significant | Significant | Total |
| --- | --- | --- | --- |
| *Staphylococcus epidermidis* | 25 | 10 | 35 |
| *Staphylococcus capitis* | 11 | 6 | 17 |
| *Staphylococcus haemolyticus* | 4 | 7 | 11 |
| *Staphylococcus hominis* | 4 | 1 | 5 |
| *Staphylococcus warneri* | 3 | 2 | 5 |
| *Staphylococcus condimenti* | 1 | 0 | 1 |
| *Staphylococcus lugdenensis* | 2 | 0 | 2 |
| *Staphylococcus saprophyticus* | 1 | 0 | 1 |
| *Staphylococcus succinus* | 1 | 0 | 1 |
| *Staphylococcus pasteuri* | 1 | 0 | 1 |
| Known | 53 | 26 | 79 |
| Unknown | 13 | 7 | 20 |
| Total | 59 | 32 | 91 |

S3 Supplementary Table 3. Coagulase Negative Staphylococci identified.
