# Supplementary material for: Neonatal bacteraemia in Ireland: A ten-year single-institution retrospective review
Source: PLoS One. 2024 Aug 23;19(8):e0306855. doi: 10.1371/journal.pone.0306855 (PMC11343407; doi:10.1371/journal.pone.0306855)
Supplement: S2 Table — (DOCX) [file pone.0306855.s004.docx]

| **Risk factors** | EONS | LONS | Not Significant | p-value |
| --- | --- | --- | --- | --- |
| N | 18 | 46 | 80 |  |
| Gest age(Days), median (IQR) | 262 (240,278) | 203(183,221) | 252(200,272) | <0.001 |
| Sex |  |  |  | 0.370 |
| F | 5 (27.8%) | 21 (45.7%) | 36 (45.0%) |  |
| M | 13 (72.2%) | 25 (54.3%) | 44 (55.0%) |  |
| Premature |  |  |  | <0.001 |
| Preterm | 9 (50.0%) | 43 (93.5%) | 49 (61.3%) |  |
| Term | 9 (50.0%) | 3 (6.5%) | 31 (38.8%) |  |
| P.R.O.M. |  |  |  | 0.400 |
| No | 12 (66.7%) | 36 (78.3%) | 65 (81.3%) |  |
| Yes | 6 (33.3%) | 10 (21.7%) | 15 (18.8%) |  |
| Maternal fever |  |  |  | 0.005 |
| No | 12 (66.7%) | 44 (95.7%) | 71 (88.8%) |  |
| Yes | 6 (33.3%) | 2 (4.3%) | 9 (11.3%) |  |
| Chorioamnionitis |  |  |  | 0.350 |
| No | 17 (94.4%) | 44 (95.7%) | 79 (98.8%) |  |
| Yes | 1 (5.6%) | 2 (4.3%) | 1 (1.3%) |  |
| Maternal UTI |  |  |  | <0.001 |
| No | 15 (83.3%) | 39 (84.8%) | 80 (100.0%) |  |
| Yes | 3 (16.7%) | 7 (15.2%) | 0 (0.0%) |  |
| P.I.C.C. |  |  |  | 0.001 |
| No | 13 (72.2%) | 22 (47.8%) | 63 (78.8%) |  |
| Yes | 5 (27.8%) | 24 (52.2%) | 17 (21.3%) |  |
| P.V.C. |  |  |  | 0.370 |
| No | 10 (55.6%) | 20 (43.5%) | 45 (56.3%) |  |
| Yes | 8 (44.4%) | 26 (56.5%) | 35 (43.8%) |  |
| U.A.C. |  |  |  | 0.690 |
| No | 14 (77.8%) | 36 (78.3%) | 67 (83.8%) |  |
| Yes | 4 (22.2%) | 10 (21.7%) | 13 (16.3%) |  |
| U.V.C. |  |  |  | 0.150 |
| No | 13 (72.2%) | 34 (73.9%) | 69 (86.3%) |  |
| Yes | 5 (27.8%) | 12 (26.1%) | 11 (13.8%) |  |
| Intravascular line |  |  |  | 0.009 |
| No lines | 5 (27.8%) | 7 (15.2%) | 33 (41.3%) |  |
| Any lines | 13 (72.2%) | 39 (84.8%) | 47 (58.8%) |  |
| ETT |  |  |  | 0.320 |
| No | 14 (77.8%) | 36 (78.3%) | 70 (87.5%) |  |
| Yes | 4 (22.2%) | 10 (21.7%) | 10 (12.5%) |  |
| TPN/Lipids |  |  |  | 0.002 |
| No | 16 (88.9%) | 31 (67.4%) | 73 (91.3%) |  |
| Yes | 2 (11.1%) | 15 (32.6%) | 7 (8.8%) |  |
| Blood transfused |  |  |  | 0.015 |
| No | 16 (88.9%) | 35 (76.1%) | 75 (93.8%) |  |
| Yes | 2 (11.1%) | 11 (23.9%) | 5 (6.3%) |  |
| Congenital abnormality |  |  |  | 1.000 |
| No | 18 (100.0%) | 45 (97.8%) | 79 (98.8%) |  |
| Yes | 0 (0.0%) | 1 (2.2%) | 1 (1.3%) |  |
| Intubation |  |  |  | 0.014 |
| No | 17 (94.4%) | 36 (78.3%) | 76 (95.0%) |  |
| Yes | 1 (5.6%) | 10 (21.7%) | 4 (5.0%) |  |

S4 Supplementary Table 4. Neonatal bacteraemia early (EONS) and late (LONS) onset by background and risk factor
