# Supplementary material for: Neonatal bacteraemia in Ireland: A ten-year single-institution retrospective review
Source: PLoS One. 2024 Aug 23;19(8):e0306855. doi: 10.1371/journal.pone.0306855 (PMC11343407; doi:10.1371/journal.pone.0306855)
Supplement: S3 Table — (DOCX) [file pone.0306855.s005.docx]

|  | | | | |
| --- | --- | --- | --- | --- |
| **Risk Factors** | **Unadjusted Odds**  **ratio**  **[95% CI] [Model 1]** | **p value** | **Adjusted Odds ratio**  **[95% CI] [Model 2]^1^** | **p value** |
| Gestational Age(days) | 0.99***(0.979,996) | 0.005 | 1.001(0.99, 1.02) | 0.897 |
| Sex |  |  |  |  |
| Female® |  |  |  |  |
| Male | 1.20(0.61, 2.33) | 0.598 |  |  |
| Premature |  |  |  |  |
| Preterm |  |  |  |  |
| Term® | 0.36***(0.17, 0.79) | 0.010 | 0.39(0.11, 1.46) | 0.162 |
| P.R.O.M. |  |  |  |  |
| No® |  |  |  |  |
| Yes | 1.44(0.65, 3.21) | 0.366 |  |  |
| Maternal fever |  |  |  |  |
| No® |  |  |  |  |
| Yes | 1.13(0.41, 3.11) | 0.817 |  |  |
| Chorioamnionitis |  |  |  |  |
| No® |  |  |  |  |
| Yes | 3.89(0.39, 38.28) | 0.245 |  |  |
| P.I.C.C. |  |  |  |  |
| No® |  |  |  |  |
| Yes | 3.07***(1.48, 6.36) | 0.003 |  |  |
| P.V.C. |  |  |  |  |
| No® |  |  |  |  |
| Yes | 1.46(0.75, 2.82) | 0.264 |  |  |
| U.A.C. |  |  |  |  |
| No® |  |  |  |  |
| Yes | 1.44(0.62, 3.34) | 0.392 |  |  |
| U.V.C. |  |  |  |  |
| No® |  |  |  |  |
| Yes | 2.27*(0.98, 5.28) | 0.057 |  |  |
| ETT |  |  |  |  |
| No® |  |  |  |  |
| Yes | 1.96(0.81, 4.77) | 0.138 |  |  |
| Intravascular lines^2^ |  |  |  |  |
| N lines® |  |  |  |  |
| Any lines | 3.04***(1.41, 6.57) | 0.005 | 2.5**(1.05, 5.94) | 0.038 |
| TPN/Lipids |  |  |  |  |
| No® |  |  |  |  |
| Yes | 3.77***(1.45, 9.79) | 0.006 | 1.58(0.49, 5.16) | 0.445 |
| Blood transfused |  |  |  |  |
| No® |  |  |  |  |
| Yes | 3.82**(1.28, 11.38) | 0.016 | 1.74(0.47, 6.48) | 0.410 |
| Ventilated |  |  |  |  |
| No® |  |  |  |  |
| Yes | 1.80(0.88, 3.68) | 0.107 |  |  |
| Congenital abnormality |  |  |  |  |
| No® |  |  |  |  |
| Yes | 1.25(0.08, 20.45) | 0.874 |  |  |
| Note: 1. In adjusted model (Model 2), we have included only those risk factors coming significant in unadjusted logistic regression model (Model 1)_ | | | | |
| 2. Intravascular lines is the combination of P.I.C.C., P.V.C., U.A.C., U.V.C., ETT and included in the adjusted model  ®: Reference category; ***: p<0.01; **: p<0.05; *:p<0.10 | | | | |

S5 Supplementary Table 5. Results of binary logistic regression unadjusted and adjusted Odds ratios of Neonatal bacteraemia by background and risk factor
