# Supplementary material for: Neonatal bacteraemia in Ireland: A ten-year single-institution retrospective review
Source: PLoS One. 2024 Aug 23;19(8):e0306855. doi: 10.1371/journal.pone.0306855 (PMC11343407; doi:10.1371/journal.pone.0306855)
Supplement: S4 Table — (DOCX) [file pone.0306855.s006.docx]

|  | | | | |
| --- | --- | --- | --- | --- |
| Risk Factors | **Adjusted Odds ratios [95% CI]** | | | |
|  | **Model 1** | **p value** | **Model 2** | **p value** |
| **Gestational age(days)** | 0.99(0.97, 1.01) | 0.370 | 0.999(0.98, 1.02) | 0.952 |
| Sex |  |  |  |  |
| Female® |  |  |  |  |
| Male | 1.30(0.61, 2.77) | 0.504 | 1.36(0.64, 2.89) | 0.419 |
| Premature |  |  |  |  |
| Preterm |  |  |  |  |
| Term® | 1.70(0.43, 6.73) | 0.447 | 2.77(0.70, 10.92) | 0.145 |
| P.R.O.M. |  |  |  |  |
| No® |  |  |  |  |
| Yes | 1.10(0.42, 2.87) | 0.845 | 1.21(0.48, 3.05) | 0.679 |
| Maternal fever |  |  |  |  |
| No® |  |  |  |  |
| Yes | 1.82(0.53, 6.23) | 0.342 | 1.34(0.39, 4.6) | 0.638 |
| Chorioamnionitis |  |  |  |  |
| No® |  |  |  |  |
| Yes | 2.10(0.14, 32.46) | 0.596 | 3.53(0.26, 48.26) | 0.345 |
| P.I.C.C. |  |  |  |  |
| No® |  |  |  |  |
| Yes | 2.05(0.82, 5.08) | 0.123 |  |  |
| P.V.C. |  |  |  |  |
| No® |  |  |  |  |
| Yes | 1.41(0.65, 3.05) | 0.380 |  |  |
| U.A.C. |  |  |  |  |
| No |  |  |  |  |
| Yes | 0.38(0.11, 1.33) | 0.130 |  |  |
| U.V.C. |  |  |  |  |
| No |  |  |  |  |
| Yes | 2.28(0.68, 7.6) | 0.180 |  |  |
| Intravascular lines^2^ |  |  |  |  |
| No lines |  |  |  |  |
| Any lines |  |  | 2.73**(1.1, 6.77) | 0.031 |
| ETT |  |  |  |  |
| No |  |  |  |  |
| Yes | 0.67(0.17, 2.57) | 0.561 |  |  |
| TPN/Lipids |  |  |  |  |
| No® |  |  |  |  |
| Yes | 1.36(0.35, 5.28) | 0.660 | 1.39(0.39, 4.92) | 0.608 |
| Blood transfused |  |  |  |  |
| No® |  |  |  |  |
| Yes | 3.14(0.58, 16.86) | 0.183 | 2.38(0.55, 10.23) | 0.245 |
| Congenital abnormality | |  |  |  |
| No® |  |  |  |  |
| Yes | 1.78(0.09, 34.14) | 0.701 | 1.97(0.11, 36.01) | 0.649 |
| Note. In model 2, we have included lines as the combination of P.I.C.C., P.V.C., U.A.C., U.V.C., ETT  ®: Reference category; ***: p<0.01; **: p<0.05; *:p<0.10 | | | | |

S6 Supplementary Table 6. Results of binary logistic regression adjusted Odds ratios of neonatal bacteraemia by background and risk factor
